# Innate immune responses to *Borrelia burgdorferi* during tick-feeding: mechanistic insights relevant to Lyme disease

**DOI:** 10.1128/mbio.03971-25

**Published:** 2026-04-20

**Authors:** Suman Kundu, Greg Joyner, Maria Gomes-Solecki

**Affiliations:** 1 Department of Microbiology, Immunology and Biochemistry, University of Tennessee Health Science Center 274062, Memphis, Tennessee, USA; 2 Flow Cytometry and Cell Sorting Core, University of Tennessee Health Science Center 12326, Memphis, Tennessee, USA; Montana State University, Bozeman, Montana, USA

**Keywords:** Lyme disease, *Borrelia burgdorferi*, tick, *Ixodes*, tick-feeding, innate immunity, myeloid cell, neutrophils, skin, chemotaxis, cytokine, mouse

## Abstract

Immune responses to tick-transmitted *Borrelia burgdorferi* (Bb) have not been characterized *in vivo*. We analyzed the reservoir host’s local and systemic immune responses to Bb during tick feeding. C3H/HeN mice were challenged via infected or uninfected nymphal *Ixodes scapularis* ticks or by subcutaneous injection of cultured multi-strain Bb. Skin and spleen tissues were evaluated by flow cytometry, serum was evaluated by cytokine proteome array, and all data were analyzed by comparison between each of the three challenged groups against the subcutaneously inoculated PBS control. Flow cytometry profiling shows that neutrophils, Langerhans cells, macrophages, B cells, and Natural Killer cells were mobilized in the three challenged groups in the skin and spleen, dendritic cells were increased in tick-transmitted groups, and T cells were engaged in Bb-challenged groups. Regarding soluble chemotaxis mediators in blood, all chemokines and cytokines induced by subcutaneously delivered Bb and uninfected tick were also induced by tick-transmitted Bb. However, tick-transmitted Bb induced unique factors absent in the other groups, which included chemokines involved in recruitment of phagocytic cells and T cell engagement, and cytokines associated with broader T cell activation. Regarding anti-inflammatory mediators, although IL-1ra was increased in the three challenged groups, IL-10 was only increased in tick-challenged groups with or without Bb. The data suggest that tick-transmitted Bb induced much more dynamic but regulated immune responses during tick-feeding, compared to subcutaneously delivered Bb, which may explain Bb persistence in the reservoir host.

**IMPORTANCE:**

Current knowledge on immune cell interactions with *Borrelia burgdorferi* (Bb) derives mostly from studies done *in vitro* and *ex vivo,* which cannot assess host immunity to natural tick-delivered Bb within the complex architecture of host tissues. We report the first *in vivo* study on local and systemic immune responses to Bb during tick feeding on a surrogate reservoir host, in comparison with uninfected-tick and subcutaneously delivered Bb. We show that uninfected-tick and tick-transmitted Bb engaged mixed type-1/type-2/type-17 immune responses in the presence of anti-inflammatory IL-10, in contrast to a type-1 response induced by subcutaneously delivered Bb. Analyses of immune responses to tick-transmitted Bb in a reservoir host can enlighten immunity mechanisms that mediate persistence of Bb.

## INTRODUCTION

After inoculation of *Borrelia burgdorferi* (Bb) in the epi/dermal layer of the skin by a nymphal *Ixodes scapularis* tick, spirochetes transit through the extracellular matrix (1, 2) where they interact with resident and recruited immune cells (3) before they disseminate widely to most tissues of the host (4, 5), oftentimes causing the pathology known to define Lyme disease. To adapt and persist in the host, unique immune evasion mechanisms are induced by Bb outer surface proteins, as well as tick saliva components that bind complement regulators (6–10), modulate chemotaxis (11–13), neutralize reactive oxygen species by neutrophils (14, 15), suppress phagocytosis (16, 17), and modulate adaptive responses by T cells (18, 19).

Although innate responses are engaged at all stages of infection, their greatest impact will be in the initial phase when Bb enters the host, replicates, and disseminates (3). Much of our understanding about innate immune responses to Bb has been derived from *in vitro* and *ex vivo* studies of interactions of cultured Bb and its outer surface components with primary immune cells or cell lines. This is a major caveat given Bb’s rapid ability to change gene expression depending on environmental cues (culture, tick, or mammal) (20), which may affect protein expression. Some innate immune cell pathways have also been investigated in mutant mice after subcutaneous (SC) inoculation of spirochetes (3). However, SC inoculation bypasses the complex host-vector-pathogen interactions that occur during natural tick transmission (20, 21). Although mice, which are surrogate reservoir hosts of Bb, do not develop the characteristic clinical pathology of Lyme disease (22), they provide important insights into immune components that are critical for limiting Bb burden, resolving inflammation, preventing infection, and identifying Bb’s key immune evasion strategies (3). While the effects of tick saliva on infection dynamics have been studied (23, 24), the impact of tick-delivered Bb on local and systemic innate immunity remains poorly understood within the three-dimensional structures of tissues *in vivo* (3, 25, 26).

The main goal of this study was to analyze the largely unknown immune cell types mobilized in mouse skin and spleen, as well as the chemotaxis mediators engaged following tick-transmitted multi-strain *B. burgdorferi* infection in comparison to responses elicited by uninfected ticks during tick feeding and needle inoculation of multi-strain Bb. Another goal was to provide a bridge for extrapolation of critical data acquired on Bb’s immune cell interactions *in vitro* and *ex vivo* with data collected from the unique *in vivo* environment in which Bb resides in the earliest stage of infection.

## RESULTS

### *B. burgdorferi* dissemination

**Fig 1:**
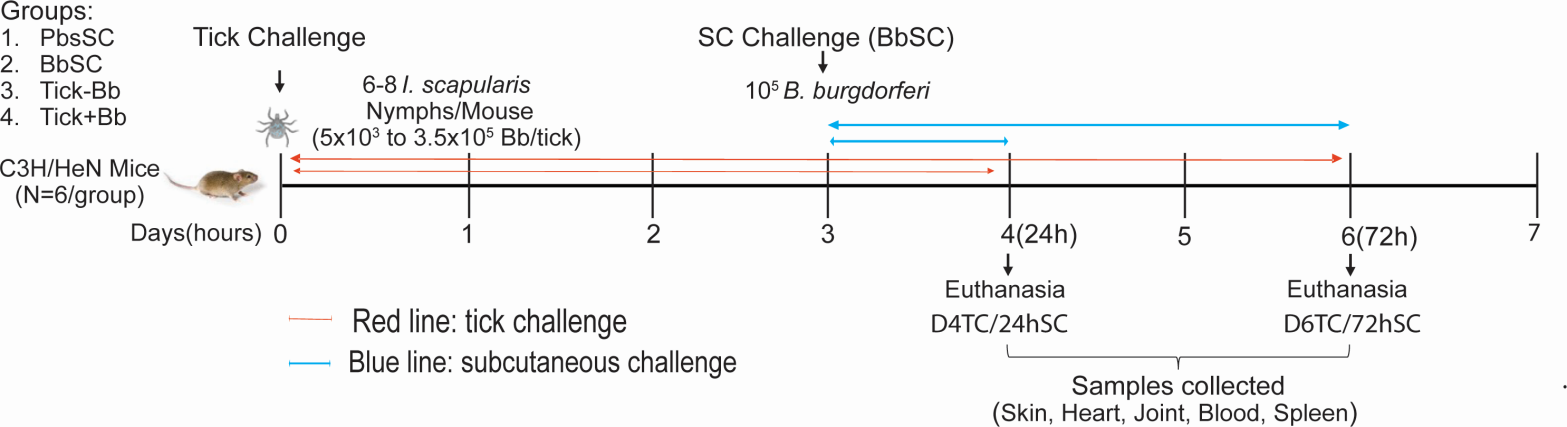
Experimental timeline and challenge schedule. Four groups of C3H/HeN mice (*n* = 3 per group) were used in two independent experiments. Group 1 (PbsSC) received a subcutaneous (SC) injection of PBS; group 2 (BbSC) was infected with 1 × 10^5^ multi-strain *B. burgdorferi* by subcutaneous injection; groups 3 (Tick–Bb) and 4 (Tick+Bb) were challenged with 6–8 nymphal *Ixodes scapularis* ticks without or with multi-strain *B. burgdorferi* (5 × 10^3^– 3.5 × 10^5^ flaB genomes/mouse). Total *n* = 6/group. Red and blue arrows indicate the time (days/hours) after the first day of tick (TC) and subcutaneous (SC) challenge: D4TC/24hSC and D6TC/72hSC.

Naïve female C3H/HeN mice (*n* = 6 per group) were challenged with 6–8 uninfected nymphal ticks (Tick–Bb) or with 6–8 ticks infected with multiple strains of *B. burgdorferi* (27) (Tick+Bb). Nymphs carried 5 × 10^3^ to 3.5 × 10^5^ Bb per tick. We used >5 nymphs per mouse to overcome differences in Bb load within ticks. Infectiousness of ticks used for challenge was confirmed in concurrent experiments using the same cohort of infected nymphs (27–29). A third group of mice received a subcutaneous injection of 10^5^ multi-strain Bb (BbSC), and a fourth group received subcutaneous PBS as a control (PbsSC). Strain composition and infectiousness of cultured Bb were determined by OspC type sequencing of DNA purified from BSK-H culture, from ticks that fed on subcutaneously infected mice, and from heart culture from infected mice (Fig. S1). Bb dissemination in tissues such as the tick-bite and SC inoculation site (skin), heart, and joint was analyzed using q-PCR at two time points: D4TC/24hSC (day 4 post-first day of tick challenge, roughly corresponding to ~24 h post-subcutaneous challenge) and D6TC/72hSC (day 6 post-first day of tick challenge, roughly corresponding to ~72 h post-subcutaneous challenge). We used D4 as the earliest TC time point based on the duration of tick attachment associated with the greatest Bb transmission to most rodents (30). D4 is also known as *big gulp* day, when ticks first appear fully engorged, many of which begin detaching from the mice, and D6 is the day when all ticks have completed their feeding and detached from the mice. An overview of the experimental layout is provided in Fig. 1.

**Fig 2:**
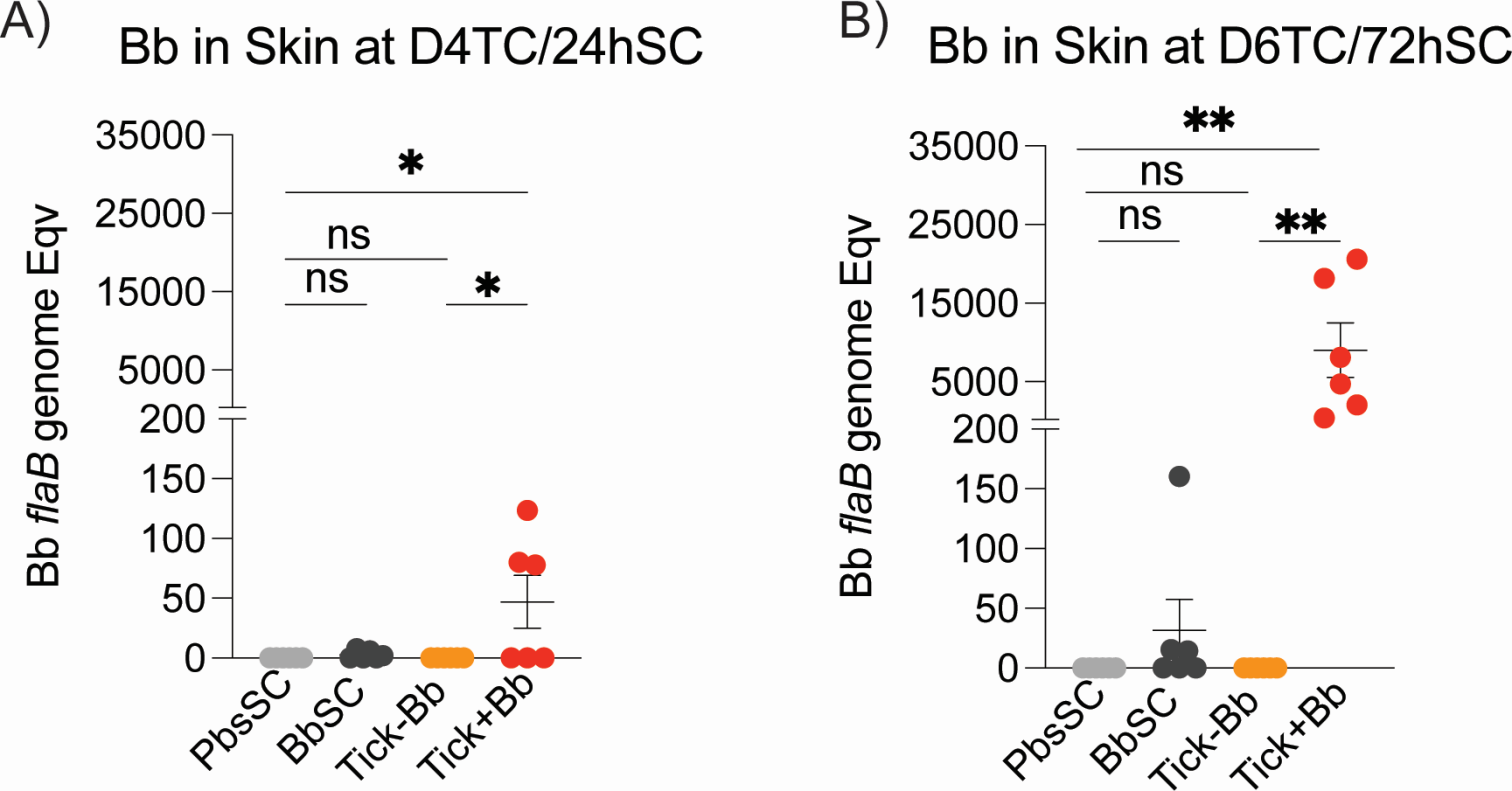
*B. burgdorferi* dissemination in the skin. Quantification of *B. burgdorferi flaB* DNA in skin at (**A**) D4TC/24hSC and (**B**) D6TC/72hSC by qPCR, *n* = 6 mice/group. Each data point represents an individual mouse; lines show group mean ± SEM. One-way ANOVA with Tukey’s post hoc test was used to compare groups (*P < 0.05, **P < 0.01; ns, not significant).

*B. burgdorferi flaB* DNA was detected in skin at the tick-bite site (Fig. 2), in 3/6 (50%) of Tick+Bb mice at day 4 post-tick challenge (D4TC) with burdens ~0–150 *flaB* genomes but not in BbSC 24 h post-infection (Fig. 2A). In addition, 6/6 (100%) Tick+Bb mice had *flaB* DNA in skin at day 6 post-tick challenge (D6TC) with burdens ranging from ~400 to 20,000 *flaB* genomes, and 1 mouse out of 6 (16.7%) BbSC had ~160 *flaB* genomes 72 h post-infection (Fig. 2B). We note that at D4TC, skin was collected from an area where most ticks were visibly engorged but had not dropped off; at D6TC, skin was collected from an area where engorged ticks had completed feeding and dropped off. No sign of Bb dissemination was observed in tissues (heart or joint) at D4 or D6 in any of the challenged groups.

### Local innate cells in skin

**Fig 3:**
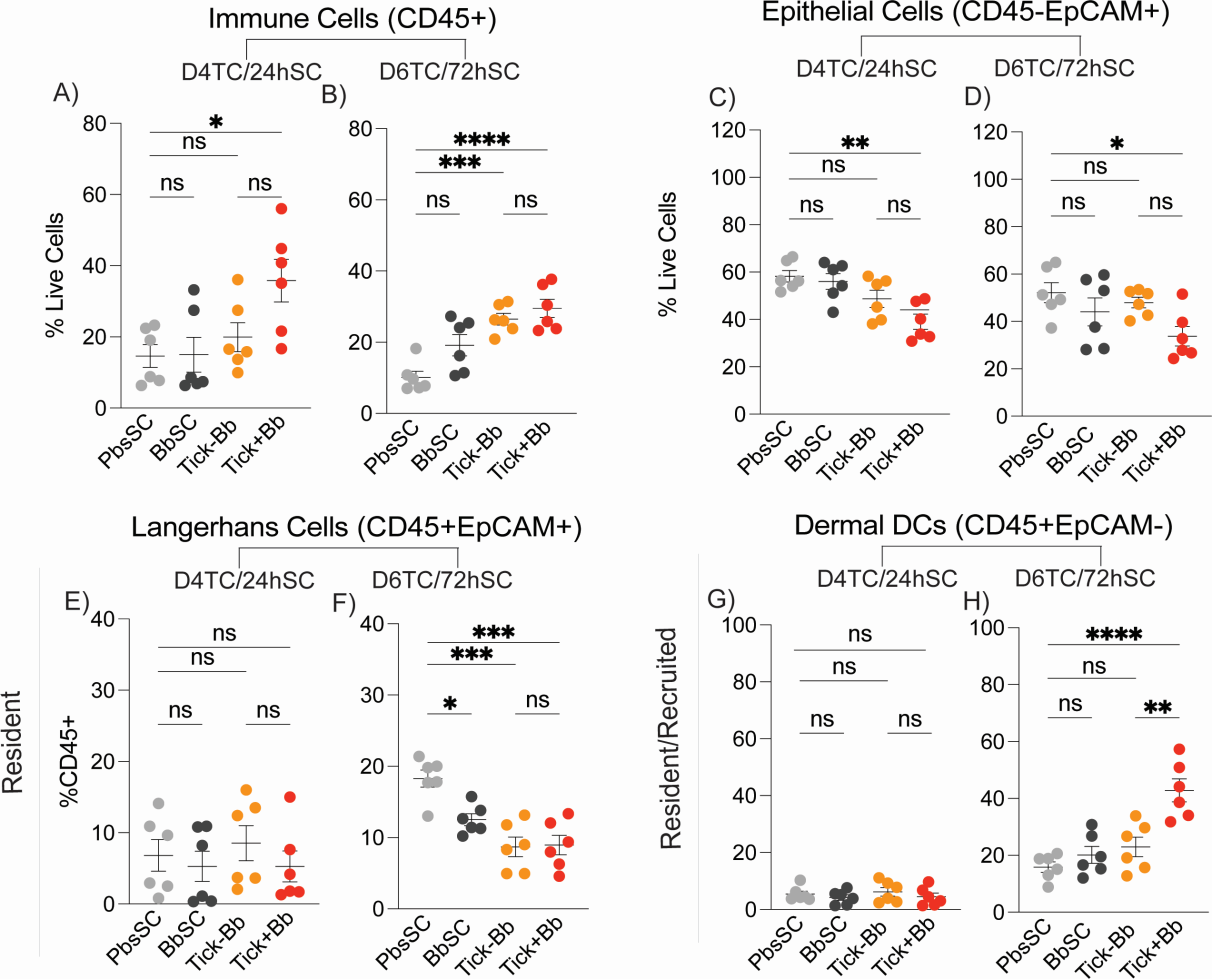
Local innate and resident cell subsets in skin. Flow-cytometric immunophenotyping of skin, *n* = 6 mice/group: (**A and B**) total immune cells (CD45^+^), (**C and D**) epithelial cells (CD45⁻EpCAM^+^), (**E and F**) Langerhans cells (CD45^+^EpCAM^+^), and (**G and H**) dermal dendritic cells (CD45^+^EpCAM⁻) at D4TC/24hSC and D6TC/72hSC, respectively, *n* = 6 per group. Each point is an individual mouse; lines indicate mean ± SEM. One-way ANOVA with Tukey’s post hoc test; *P < 0.05, **P < 0.01, ***P < 0.001, ****P < 0.0001; ns, not significant.

**Fig 4:**
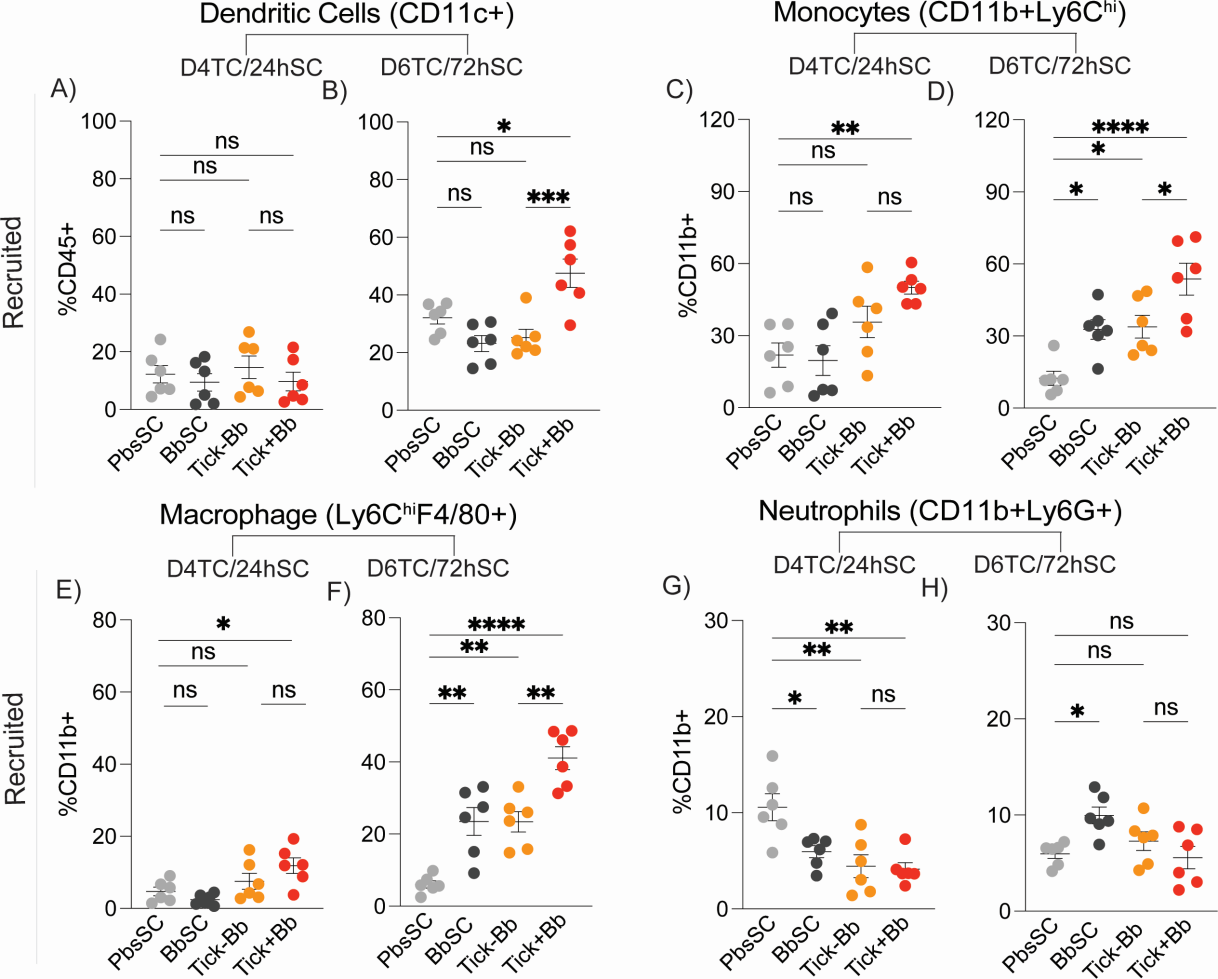
Recruited myeloid innate cell subsets in skin. Flow-cytometric analysis of recruited immune cells, *n* = 6 mice/group: (**A and B**) dendritic cells (CD11c^+^), (**C and D**) monocytes (CD11b^+^Ly6C^hi^), (**E and F**) macrophages (Ly6C^hi^F4/80^+^), and (**G and H**) neutrophils (CD11b^+^Ly6G^+^) at D4TC/24hSC and D6TC/72hSC. Each point is an individual mouse; lines indicate mean ± SEM. One-way ANOVA with Tukey’s post hoc test; *P < 0.05, **P < 0.01, ****P < 0.0001, ***P *<* 0.001; ns, not significant.

To examine cellular immune responses at the Bb inoculation site, 1 cm² of skin was collected after challenge from either the tick-bite site at D4TC and D6TC, or from the needle-inoculated site at 24hSC and 72hSC and processed into single-cell suspensions for flow-cytometric immunophenotyping. Results from the two time points, D4TC/24hSC and D6TC/72hSC, are presented in Fig. 3 and 4. Figure 3 shows proportions of live innate CD45^+^ immune cells (Fig. 3A and B), epithelial cells CD45⁻EpCAM^+^ (Fig. 3C and D), skin-resident Langerhans CD45^+^EpCAM^+^ (Fig. 3E and F) and resident/recruited dermal dendritic cells (DCs) CD45^+^EpCAM⁻ (Fig. 3G and H). Figure 4 shows the recruited migratory innate populations of dendritic cells CD45^+^CD11c^+^ (Fig. 4A and B), monocytes CD11b^+^Ly6C^hi^ (Fig. 4C and D), macrophages CD11b^+^Ly6C^hi^F4/80^+^ (Fig. 4E and F), and neutrophils CD11b^+^Ly6G^+^ (Fig. 4G and H). Comparisons were done between the naïve control PbsSC and each of the challenged groups (BbSC, Tick–Bb, Tick+Bb), and between TC (Tick−Bb vs Tick+Bb) groups.

The strongest differences in innate myeloid immune cell populations were generally measured at D6TC/72hSC, except for epithelial cells and neutrophils, with strongest differences at D4TC/24hSC. Furthermore, at D4TC/24hSC, there were no differences between the experimental groups and naïve control in resident Langerhans cells (Fig. 3E), resident dermal DCs (Fig. 3G), and recruited DCs (Fig. 4A). This could be associated with stronger evidence for skin colonization by Bb at D6TC/72hSC (Fig. 2). Specifically, CD45^+^ leukocyte infiltration was greater in Tick+Bb than PbsSC at D4TC/24hSC, as well as in Tick+Bb and Tick–Bb versus PbsSC at D6TC/72hSC, with no differences measured between the SC or TC groups (Fig. 3A and B). Epithelial cells were reduced in Tick+Bb but not in Tick–Bb compared with PbsSC, with no differences measured between SC and TC groups at both time points (Fig. 3C and D). Resident Langerhans cells (Fig. 3F) were reduced in Tick+Bb, Tick–Bb, and BbSC relative to PbsSC with no differences measured between TC groups at D6TC/72hSC (Fig. 3F). Dermal dendritic cells (DDCs), which include resident and recruited subsets, were increased in Tick+Bb relative to PbsSC and Tick−Bb, with no differences measured between SC groups at D6TC/72hSC (Fig. 3H). In summary (Fig. 3), Tick+Bb led to the strongest differences in recruited and resident immune cell populations in the skin, with tick factors (Tick–Bb) assisting independently in the infiltration of CD45^+^ leucocytes and reduction of resident Langerhans cells (LCs CD45^+^EpCAM^+^), but not affecting epithelial cells (EC, CD45⁻EpCAM^+^) or dermal DCs (CD45^+^EpCAM⁻). Reductions of resident Langerhans cells (LCs CD45^+^EpCAM^+^) were also affected by BbSC at D6TC/72hSC.

Regarding recruited migratory innate cell subsets in the skin (Fig. 4) at D6TC/72hSC, dendritic cell populations (Fig. 4B) were increased in Tick+Bb relative to PbsSC and Tick–Bb, with no differences measured between SC groups, which mirrors dermal DCs (DDCs, Fig. 3H); monocytes were increased in Tick+Bb, Tick–Bb, and BbSC compared with PbsSC, and increases between TC groups were also significant at D6TC/72hSC (Fig. 4D); the same trend was observed for Tick+Bb and PbsSC at D4TC/24hSC (Fig. 4C); as expected, macrophages (Fig. 4E and F) maintained the same pattern as monocytes (Fig. 4C and D). Regarding neutrophil populations (Fig. 4G and H), they were decreased in Tick+Bb, Tick–Bb, and BbSC compared to PbsSC, and differences between TC groups were not significant at D4TC/24hSC (Fig. 4G). Strikingly, at D6TC/72hSC (Fig. 4H), neutrophil populations were equivalent in Tick+Bb and Tick–Bb versus the PbsSC but increased in BbSC versus PbsSC. In summary (Fig. 4), Tick+Bb led to the strongest increases in dendritic cells (DCs), monocytes, and macrophages in the skin, with tick factors (Tick–Bb) and Bb (BbSC) contributing independently to differences in monocytes (CD11b^+^Ly6 C^hi^) and macrophages (CD11b^+^Ly6C^hi^F4/80^+^), but not to the recruitment of dendritic cells (DC, CD45^+^CD11c^+^). Regarding neutrophils (CD11b^+^Ly6G^+^), tick-transmitted Bb (Tick+Bb), tick factors (Tick–Bb), and Bb (BbSC) contributed independently to reductions of recruited neutrophils at D4TC/24hSC; at D6TC/72hSC, only mice challenged SC with Bb (BbSC) had increased populations of neutrophils in the skin relative to PbsSC.

### Cellular responses in spleen

**Fig 5:**
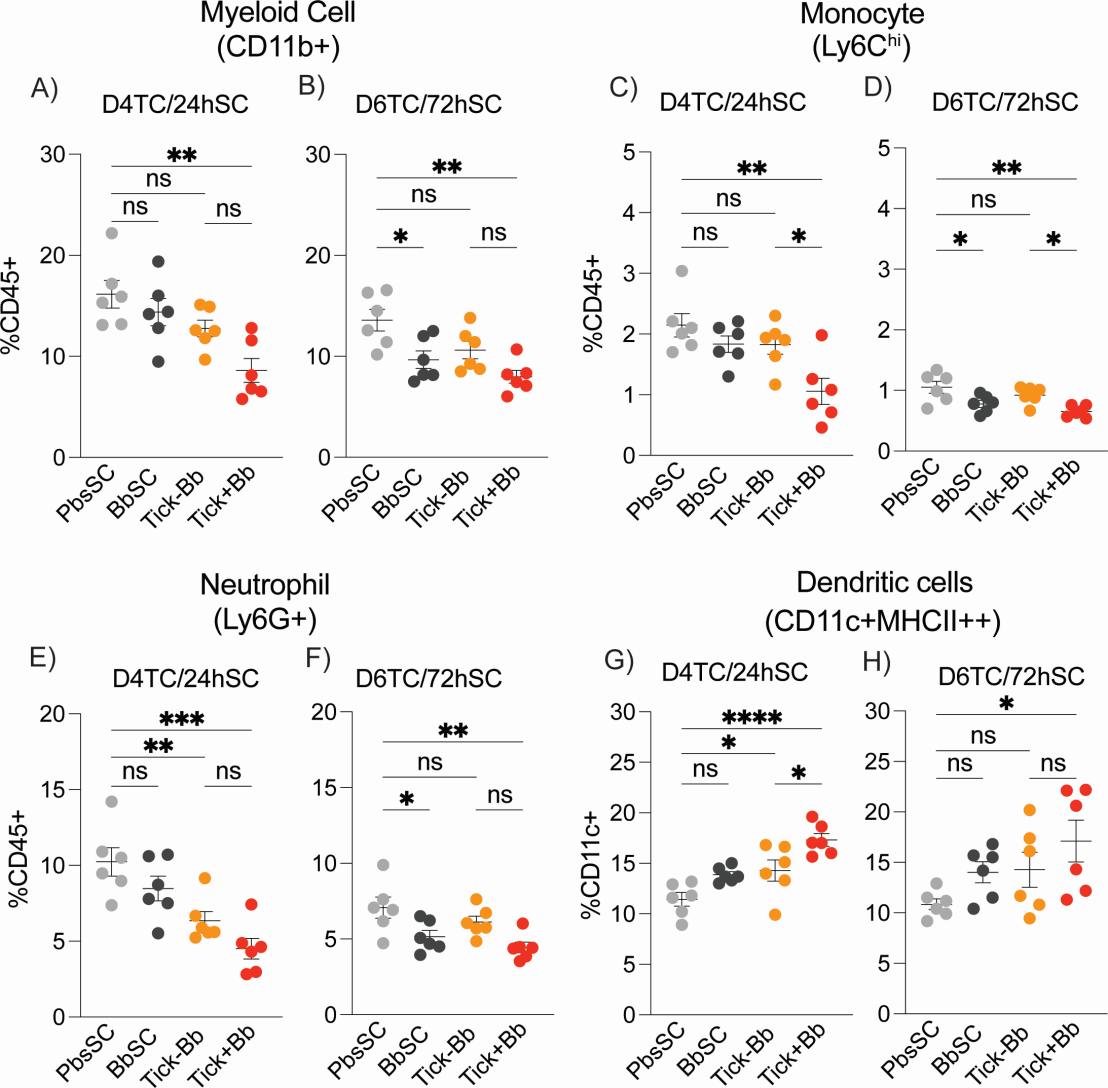
Myeloid cells in spleen. Flow-cytometric immunophenotyping of splenocytes: (**A and B**) myeloid cells (CD11b^+^), (**C and D**) monocytes (Ly6C^hi^), (**E and F**) neutrophils (Ly6G^+^), and (**G and H**) dendritic cells (CD11c^+^MHCII^+^) at D4TC/24hSC and D6TC/72hSC, *n* = 6 mice per group. Each point is an individual mouse; lines indicate mean ± SEM. One-way ANOVA with Tukey’s post hoc test; *P < 0.05, **P < 0.01, ***P < 0.001, ****P < 0.0001; ns, not significant.

**Fig 6:**
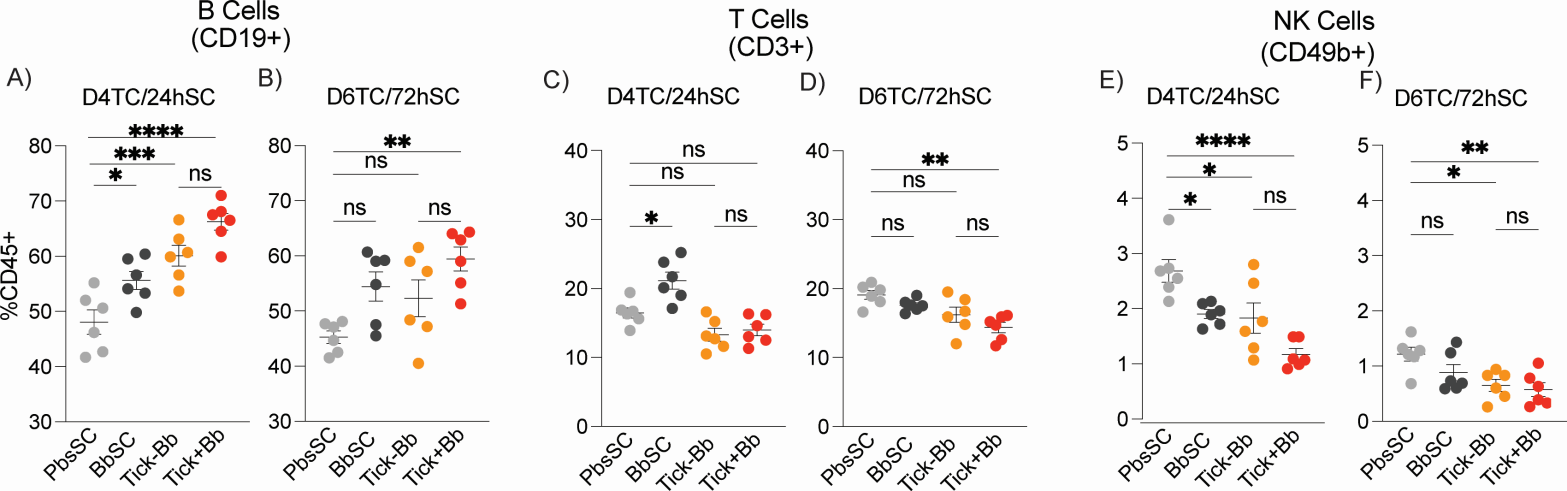
Lymphoid cells in spleen. Flow-cytometric immunophenotyping of splenocytes: (**A and B**) B cells (CD19^+^), (**C and D**) T cells (CD3^+^), (**E and F**) NK cells (CD49b^+^) at D4TC/24hSC and D6TC/72hSC, *n* = 6 mice/group. Each point is an individual mouse; lines indicate mean ± SEM. One-way ANOVA with Tukey’s post hoc test; *P < 0.05, **P < 0.01, ***P < 0.001, ****P < 0.0001; ns, not significant.

Systemic myeloid and lymphoid immune cell profiles were assessed in the spleen, a secondary lymphoid organ, by flow-cytometric immunophenotyping at D4TC/24hSC and D6TC/72hSC (Fig. 5 and 6). In contrast to the skin, most splenic immune cell differences were evident at D4TC/24hSC, and the same trends were observed at D6TC/72hSC. Regarding the myeloid germline (Fig. 5), myeloid CD11b^+^ cells (Fig. 5A and B), Ly6C^hi^ monocytes (Fig. 5C and D), and Ly6G^+^ neutrophils (Fig. 5E and F) were reduced in Tick+Bb versus PbsSC at both time points, whereas the CD11c^+^MHCII^+^ dendritic cells were increased (Fig. 5G and H). Tick factors (Tick–Bb) independently contributed to differences in Ly6G^+^ neutrophils (Fig. 5E) and CD11c^+^MHCII^+^ dendritic cells (Fig. 5G) at D4TC/24hSC, whereas BbSC contributed to reductions in myeloid CD11b^+^ (Fig. 5B), Ly6C^hi^ monocytes (Fig. 5D), and Ly6G^+^ neutrophils (Fig. 5F) at D6TC/72hSC. Differences between tick-challenged groups (Tick+Bb and Tick–Bb) were also significant for monocytes (Fig. 5C and D) and dendritic cells (Fig. 5G).

Regarding the lymphoid germline (Fig. 6), CD19^+^ B cell populations were increased in Tick+Bb, Tick–Bb, and BbSC versus the PbsSC at D4TC/24hSC (Fig. 6A), whereas CD94b^+^ NK cells were decreased (Fig. 6E). Similar trends were observed at D6TC/72hSC (Fig. 6B and F). CD3^+^ T cells were increased in the BbSC group relative to PbsSC at D4TC/24hSC (Fig. 6C), whereas they were decreased in the Tick+Bb group relative to PbsSC at D6TC/72hSC (Fig. 6D). Tick factors (Tick–Bb) independently contributed to differences in B cells and NK cells, but not in T cells, whereas BbSC contributed to differences in all three cell types.

In summary (Fig. 5 and 6), Tick+Bb led to the strongest differences in myeloid and lymphoid cell populations in the spleen, which were aided by tick factors (Tick–Bb) in neutrophils, dendritic cells, B cells, and NK cells but not in monocytes and T cells. Of note, needle inoculation of Bb led to significant reductions of populations of the myeloid line (monocytes, neutrophils) except dendritic cells at (D6TC/72hSC), whereas it caused significant increases in populations of the lymphoid line at D4TC/24hSC (B cells, T cells, and NK cells).

### Serum chemokine responses

**Fig 7:**
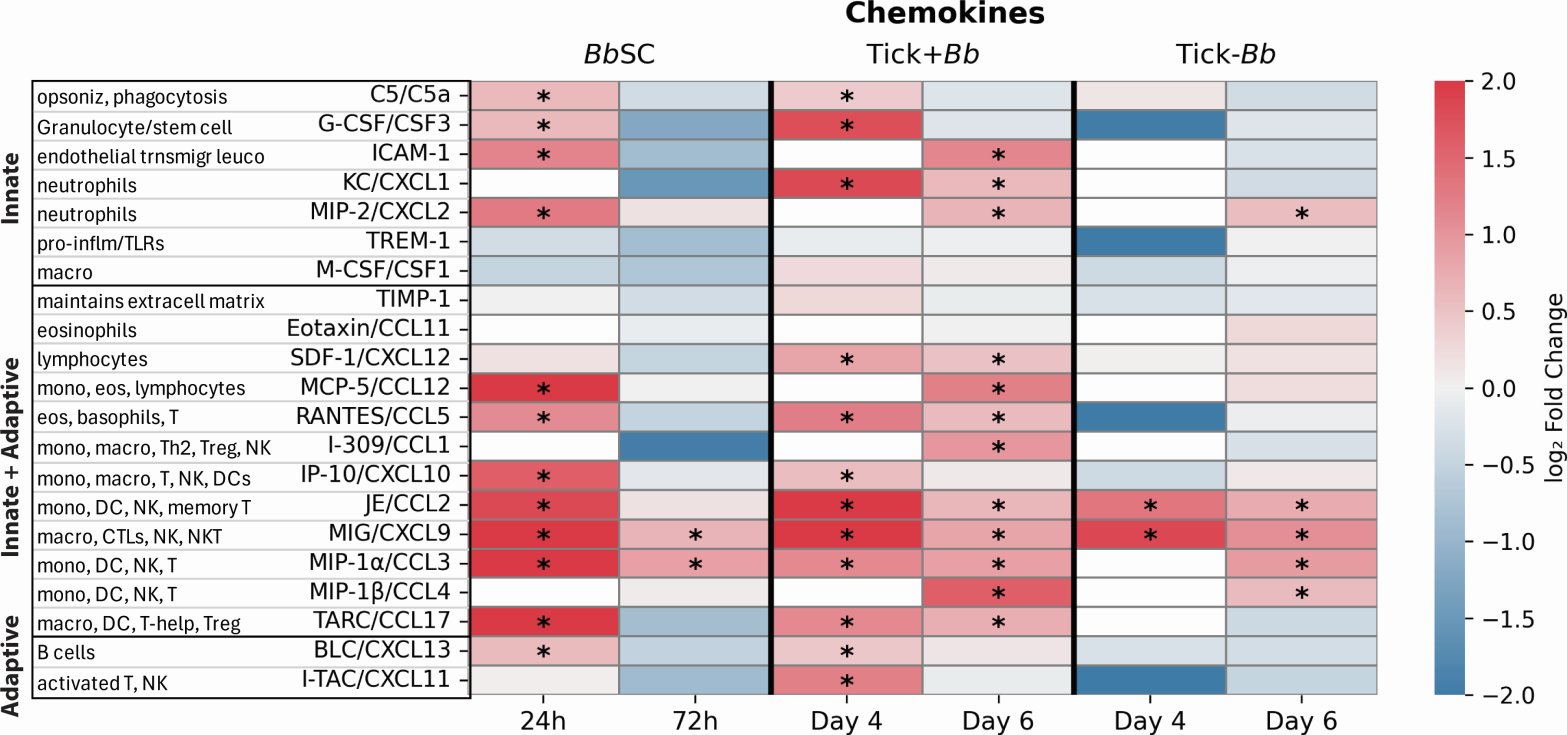
Chemokines in mouse serum. Heatmap of log₂ fold change relative to pooled PbsSC controls (capped at ±2) for C5/C5a and 20 chemokines measured with the Proteome Profiler Mouse Cytokine Array. Serum from each group (pooled *n* = 3 mice) was analyzed at D4TC/24hSC (left panels) and D6TC/72hSC (right panels) for the three challenge conditions: multi-strain *B. burgdorferi* subcutaneous inoculation (BbSC), uninfected tick feeding (Tick–Bb), and multi-strain *B. burgdorferi*-infected tick feeding (Tick+Bb). Color scale ranges from blue (down-regulation) to red (up-regulation); each square shows the log₂ fold change of the mean signal from duplicate capture spots relative to the PbsSC control. * denotes significant increase by cutoff >0.32 log_2_ fold change, equivalent to 1.25 fold change.

Twenty (20) circulating chemokines and complement factor C5/C5a were quantified using the Mouse Proteome Profiler Cytokine Array (R&D Systems, ARY006) according to the manufacturer’s instructions (Fig. 7). Mean pixel density of duplicate capture spots for each analyte was calculated with QuickSpots software (Ideal Eyes System, USA). Serum samples from experimental mice were analyzed at D4TC/24hSC and D6TC/72hSC, and results are expressed as fold change for the challenged groups (BbSC, Tick–Bb, Tick+Bb) relative to the uninfected control (PbsSC). Significant differences were determined at cutoff >0.32 log_2_ fold change (~1.25 fold change). Bb inoculated SC (BbSC) and tick-transmitted Bb (Tick+Bb) induced complement factor C5/C5a. BbSC induced secretion of 11/20 (55%) chemokines in serum at D4TC/24hSC, and 2 of these remained increased at D6TC/72hSC. Tick+Bb induced secretion of 11/20 chemokines in serum at D4TC/24hSC, and 5 additional chemokines were increased at D6TC/72hSC for a total of 16/20 (80%). Uninfected tick (Tick–Bb) induced secretion of 2/20 chemokines at D4TC/24hSC, and 3 additional chemokines were increased at D6TC/72hSC for a total of 5/20 (25%). Of note, all chemokines induced by BbSC and Tick–Bb were also induced by Tick+Bb. The following four chemokines were uniquely induced by Tick+Bb: KC/CXCL1, SDF-1/CXCL12, I-309/CCL1, and I-TAC/CXCL11.

### Serum cytokine responses

**Fig 8:**
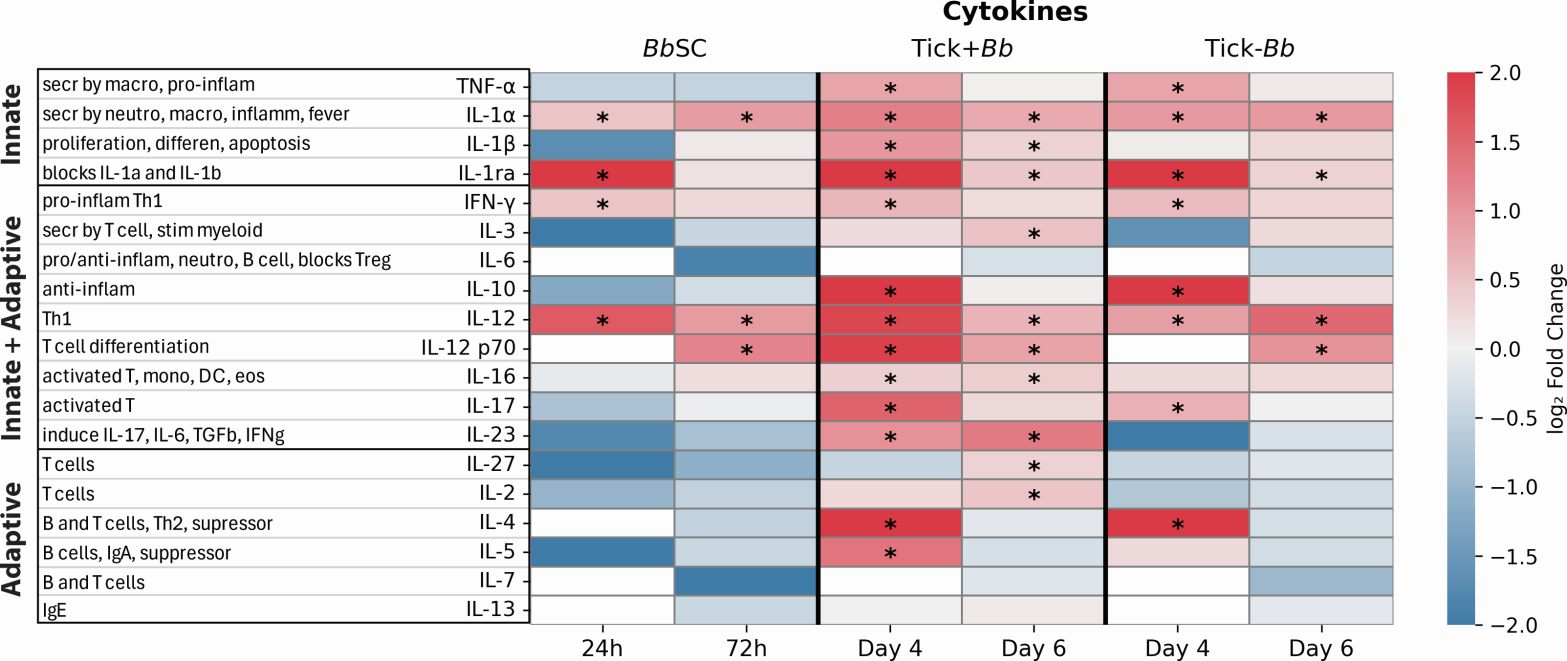
Cytokines in mouse serum. Heatmap of log₂ fold change relative to pooled PbsSC controls (capped at ±2) for 19 cytokines measured using the same array platform and experimental design as in Fig. 7. Blue indicates down-regulation and red indicates up-regulation compared with PbsSC control. * denotes significant increase by cutoff >0.58 log_2_ fold change, equivalent to 1.5 fold change.

Nineteen (19) cytokines were quantified and visualized as heatmaps to compare expression in the experimental groups (BbSC, Tick–Bb, Tick+Bb) relative to the uninfected control (PbsSC) (Fig. 8). Bb inoculated SC (BbSC) induced secretion of 4/19 cytokines in serum at D4TC/24hSC, whereas 1 additional cytokine was increased at D6TC/72hSC for a total of 5/19, 26%. Tick-transmitted Bb (Tick+Bb) induced secretion of 13/19 cytokines in serum at D4TC/24hSC, whereas 3 additional cytokines were increased at D6TC/72hSC for a total of 16/19 (84%). Uninfected tick (Tick–Bb) induced secretion of 8/19 cytokines at D4TC/24hSC, and 1 additional cytokine was increased at D6TC/72hSC for a total of 9/19 (47%). Of note, all cytokines induced by BbSC and Tick–Bb were also induced by Tick+Bb. The following seven cytokines were uniquely induced by Tick+Bb: IL-1β, IL-3, IL-16, IL-23, IL-27, IL-2, and IL-5.

**Fig 9:**
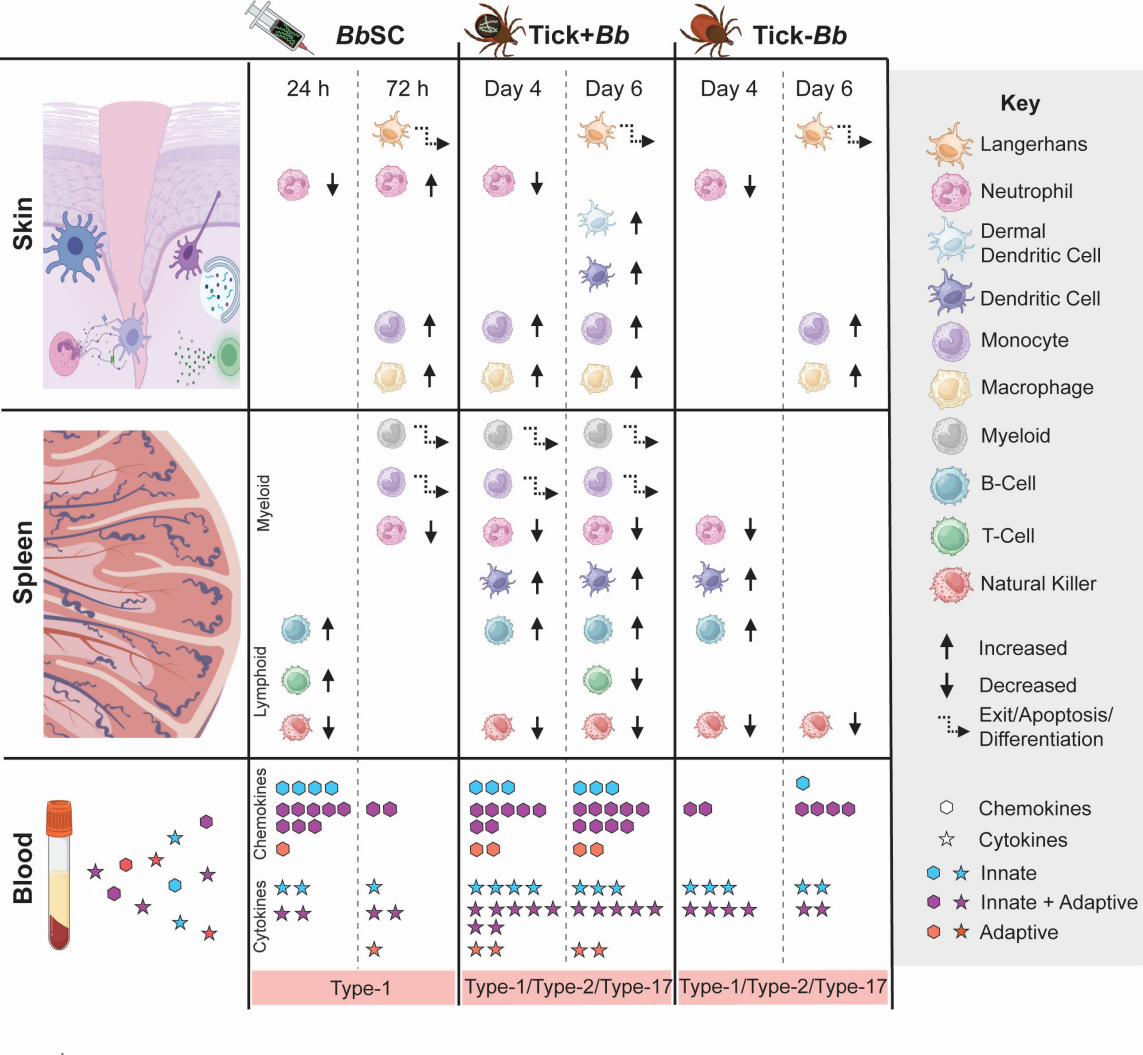
Model of murine host immune responses to Bb, tick factors, and tick-transmitted Bb relative to the control. Graphical summary of the study showing the infection conditions—subcutaneous needle inoculation of multi-strain Bb (BbSC), uninfected tick feeding (Tick–Bb), and multi-strain Bb-infected tick feeding (Tick+Bb)—in C3H/HeN mice, with sequential analyses, including flow-cytometric profiling of immune cell populations at the bite/inoculation site and in spleen, and serum proteome profiling to quantify 19 cytokines and 20 chemokines.

Consistent with the splenic cells data, chemokine and cytokine responses were higher at D4TC/24hSC than at D6TC/72hSC (Fig. 7 and 8). A model of our findings is presented in Fig. 9.

## DISCUSSION

Transmission of *Borrelia burgdorferi* (Bb) via the tick bite into the skin of the host places the spirochete in an environment enriched with immune cells that promote innate and adaptive immunity (31). However, the causative agent of Lyme disease is highly adept at modulating innate immunity and evading antibody- and T cell-mediated responses (18, 32). We compared innate immune cell populations in the skin and spleen of mice, and chemotaxis mediators circulating in blood, following tick-transmitted Bb infection in comparison to uninfected tick and subcutaneous delivery of cultured infectious Bb. The ticks used for challenge were infected with 19 strains of *B. burgdorferi* as defined by OspC type (27), which engages an immune response that is more representative of what might be encountered in naturally acquired infections. We found that tick-transmitted Bb (Tick+Bb) induced the strongest differences in recruited and resident immune cell populations in skin (Fig. 3 and 4) and spleen (Fig. 5 and 6), as well as the highest number of chemotaxis mediators (Fig. 7 and 8). Specifically, the inflammatory response following tick transmission of Bb engaged mixed type-1/type-2/type-17 immune responses, which were also engaged by uninfected ticks, whereas subcutaneously delivered Bb engaged a type-1 immune response. A model is presented in Fig. 9.

Tick-transmitted *B. burgdorferi* (Tick+Bb) led to significantly higher burdens of Bb in skin at 4DTC (50%) and 6DTC (100%) than BbSC at 24 h (0%) and at 72 h (17%) post-inoculation, with numbers of Bb *flaB* genomes in skin 100× higher at 6DTC than 4DTC (Fig. 2). Furthermore, Bb *flaB* genomes were not found in heart or joint tissues. Absence of Bb in heart and joints at D4TC/24hSC and D6TC/72hSC is expected as it takes at least ~7–10 days for colonization to be established (1). Several studies have shown that tick saliva can enhance infectivity of Bb and benefit Bb transmission (10, 21, 33–35) by modulating immune responses permissive of dissemination (36). Notwithstanding limitations of qPCR detection of Bb DNA at early time points, it was surprising to not find Bb in skin of infected mice, at 24 h and at 72 h post-subcutaneous inoculation (BbSC) of a high dose of Bb. We used a high infectious dose (~10^5^ Bb) to normalize infection of SC and TC mice. Absence of Bb in the skin of SC-inoculated mice cannot be explained by Bb not being infectious, given that the culture was verified for morphology and motility by dark field microscopy (37, 38) before inoculation, and was checked for infectivity in mice (Fig. S1). Furthermore, here we report striking innate myeloid and NK, and adaptive B and T cell responses to BbSC.

Antigen-presenting cells (APCs) such as Langerhans cells (LC), DDC, DC, and macrophages recognize pathogens via PRRs, mature, and migrate to the draining lymph nodes, where they orchestrate adaptive lymphoid T, B, and NK responses that will result in the production of specific antibodies by B cells to control Bb dissemination (3, 31). All differentiated innate cells tested, resident CD45^+^EpCAM⁻ DDC and CD45^+^EpCAM^+^ LC, as well as recruited CD45^+^CD11c^+^ DC, recruited CD11b^+^Ly6C^hi^F4/80^+^ macrophages, CD11b^+^Ly6G^+^ neutrophils, and CD49b^+^ NK cells have phagocytic/APC functions and can kill Bb *in vitro* (3, 16, 39).

In skin, only tick-transmitted *B. burgdorferi* (Tick+Bb) led to a reduction of resident non-phagocytic epithelial cells (ECs) (Fig. 3C and D), which can be explained by the disruption of the epithelial barrier by the tick’s mouth parts during attachment. Reduction of resident LCs (Fig. 3F) in the skin of the three groups of challenged mice strongly suggests that LCs executed phagocytosis and antigen presentation, migrated to the local draining lymph nodes to further execute APC functions, or underwent apoptosis (40–42), possibly independently of Bb infection (21). Reductions in LC were also found in human tick bite skin (21). Others have shown that intradermal inoculation of Bb in an *ex vivo* human skin model drove migration of Langerhans cells out of the skin (43).

Dendritic cells (DCs) play an instrumental role in bridging innate cell and T cell adaptive immunity and are particularly important in defining the fate of Bb within the dermis at the earliest time points of infection (31). Dermal dendritic cells (DDCs, Fig. 3H) and dendritic cells (DCs, Fig. 4B) are only increased in skin after tick-transmitted Bb infection (Tick+Bb), but not in uninfected tick (Tick–Bb) and in subcutaneously delivered Bb (BbSC). The data suggest that during tick feeding, DC populations are enriched in the skin (possibly to cover for the exit of LCs) to continue phagocytic and APC functions. Increased CD45^+^ infiltrates are associated with tick-driven recruitment in human skin after tick bites (44). Some studies reported mature DDCs were enriched in erythema migrans lesions of Lyme disease patients (45–47). These studies are consistent with our observations in mice. Others found DCs enriched in human skin biopsies of tick bites from people not infected with Bb (44), which differs from the result we observed in mice (Tick–Bb, Fig. 4B). Regarding DC function, tick saliva inhibits DC functions like phagocytosis (48), migration to lymph nodes (49), maturation markers which aid in T cell activation, cytokine production (50), and polarization of T-cells (47, 49, 51). Although we did not measure a tick saliva effect on DC/DDC (Tick–Bb) populations in skin (Fig. 3H and 4B), the fact that it may inhibit DC migration to lymph nodes may have contributed to enrichment of DC/DDC in skin after tick-transmitted Bb (Tick+Bb) infection.

Monocytes and macrophages were also increased in skin after tick-transmitted Bb infection (Tick+Bb), as well as uninfected tick (Tick–Bb) and BbSC (Fig. 4C through F). In the spleen, myeloid cells (Fig. 5A and B), and monocytes (Fig. 5C and D), which are macrophage and DC precursors, were reduced in tick-delivered Bb groups (Tick+Bb), which is opposite to the increased mono/macrophage/DC populations measured in skin (Fig. 4). These precursors (Fig. 5A through D) may have migrated from the spleen to infiltrate skin seeded by tick-delivered Bb (Tick+Bb) or they could have differentiated into DCs as the latter are increased in spleen (Fig. 5G and H). In both cases, the function of these cells is phagocytic/APC and inflammatory chemotaxis. It has been shown that Bb disseminates through lymphatic ducts (52) in addition to migrating through the skin. Although not measured in this study, the potential presence of Bb in spleen could have explained increased populations of DCs (Fig. 5G and H), as well as the reduction of neutrophils (Fig. 5E and F) due to apoptosis (41) after execution of their function.

Neutrophils form the first line of defense against pathogens and sterile injuries (53), have been shown to kill Bb *in vitro* (39), and extrude NETs to entrap and kill Bb *in vivo* in a mouse model of tick-transmitted borreliosis (54). In skin (Fig. 4G and H and Fig. 9), we measured a reduction in neutrophil populations after challenge with the three experimental groups (Tick+Bb, Tick–Bb, and BbSC) at D4TC/24hSC (Fig. 4G) and an increase only in the needle-inoculated Bb group at D6TC/72hSC (Fig. 4H). Xu et al. (55) showed that needle-inoculation of Bb into the dermis of mice induced infiltration of neutrophils within 6 h, but the inflammatory response became macrophage-dominant by 16 h. Thus, opposite abundances of neutrophils in skin at 24hSC versus 72hSC could reflect apoptosis (41) at 24 h after these cells resumed their clearance functions, whereas increases at 72 h suggest that a new population of neutrophils subsequently entered the skin to kill persisting or replicating Bb. The data suggest that tick-transmitted Bb suppresses continued recruitment of neutrophils into the feeding pit. Suppression of neutrophils and Bb inhibition of human neutrophil functions was shown by others (56, 57). These factors may help explain the absence of neutrophils in human erythema migrans histopathology (58).

Regarding lymphoid populations in the spleen (Fig. 6), CD19^+^ B cells were increased in the three experimental groups (Tick+Bb, Tick–Bb, and BbSC) relative to the PbsSC (Fig. 6A). A strong production of *B. burgdorferi* antigen-specific antibody has long been considered evidence of robust B cell responses against Bb infection (32), although these responses are often insufficient to control Bb dissemination. CD49b^+^ Natural Killer (NK) populations, which play an important role during early host defense (59), were decreased in the three challenged groups versus PbsSC (Fig. 6E). This is consistent with the decreases observed for the other innate immune cells in the spleen, except DCs (Fig. 5G and H) that may have infiltrated this secondary lymphoid organ as APC to present antigen to B/T cells or may have differentiated from the resident myeloid cells in spleen. This suggests that NKs may have migrated to the skin or other sites of Bb persistence. Lastly, CD3^+^ T cell populations were decreased in Tick+Bb versus PbsSC (Fig. 6D), whereas T cells were increased in BbSC versus PbsSC (Fig. 6C), suggesting that tick factors may suppress Bb*-*driven T-cell activation and proliferation as shown by others (60, 61). This is, however, somewhat contradicted by the chemotaxis cross-talk circulating in blood as described below.

Regarding chemotaxis (Fig. 7 and 8), all chemokines and cytokines induced by BbSC and Tick–Bb were also induced by Tick+Bb. Of note, the following chemokines (KC/CXCL1, SDF-1/CXCL12, I-309/CCL1, and I-TAC/CXCL11) and cytokines (IL-1β, IL-3, IL-16, IL-23, IL-27, IL-2, and IL-5) were uniquely induced by Tick+Bb. The Tick+Bb-specific chemokines are involved in the recruitment of neutrophils and other myeloid cells, Th2, Treg, NK, and activated T, whereas most of the unique cytokines are involved in adaptive T cell activation. The chemotaxis factors increased in tick-transmitted Bb (Tick+Bb) engaged mixed T helper responses of the type-1, type-2, and type-17, which was supported by tick factors (Tick–Bb). In contrast, subcutaneously delivered Bb engaged a type-1 immune response. Interestingly, others found that human PBMCs stimulated *in vitro* with 15 strains of *B. burgdorferi sensu stricto* isolated from erythema migrans lesions from patients in the United States secreted chemokines and cytokines associated with innate and Th1-adaptive immune responses, and that US strains were associated with more severe disease (62). They also found that European strains of *B. burgdorferi sensu stricto*, which cause less severe disease, induced greater Th17-associated responses. Thus, we speculate that a broader T cell involvement in hosts infected by tick-transmitted Bb may represent a more regulated and balanced (rather than suppressed) immune response that allows Bb to persist in the reservoir host and enable the enzootic cycle of the spirochete.

In addition, we note that cultured spirochetes used for SC inoculation predominantly express OspA and have lower OspC expression under *in vitro* conditions, whereas spirochetes within feeding ticks and during early mammalian infection downregulate OspA and strongly upregulate OspC (63, 64). Thus, the antigens expressed on the surface of Bb at the time of host entry likely played a role in the underlying differences in immune responses reported in this study. Another factor to consider with SC-inoculated spirochetes is that there may be greater spirochete death early on for reasons beyond just surface-exposed antigens, as host adaptation requires global changes associated with RpoS activation (65, 66), which could make Bb more susceptible to killing mediated by immune cells.

It is known that Bb carrying different types of OspC/RST vary in their disseminating capabilities in humans (62, 67, 68) and that genotypic variation of Bb affects pathogenesis in C3H mice (69, 70) and humans (71). However, all these studies analyzed single isolates of cultured Bb. Although it is possible that Bb variability within ticks may affect immune responses, our study was not designed to answer that question. Our goal was to query immune responses to tick-transmitted Bb using ticks infected with multiple strains to closely mimic a natural infection of a surrogate reservoir host for which all Bb strains are expected to disseminate or else exit the enzootic cycle. We checked by OspC type sequencing that the ticks used in this study contained the 19 Bb strains reported (27), including the types most frequently found in human disseminated infections (72), and that all Bb types present in the multi-strain culture used for subcutaneous inoculations disseminated to tissues of infected mice (Fig. S1).

In conclusion, the data suggest that tick transmission of Bb led to dynamic responses by myeloid and lymphoid immune cells which lacked in neutrophil function in the skin, and resulted in a very strong presence of chemotactic factors in blood during tick feeding. These factors are usually involved in activation of T and NK cells (I-TAC/CXCL11), Th1 (IL-12), Th2 (I-309/CCL1, IL-4, IL5), and Th17 (IL-17/IL-23) representing mixed type-1/type-2/type-17 T helper responses, also present in the uninfected tick. In contrast, a more nuanced response to SC inoculation of Bb was mediated by less myeloid cell involvement with productive engagement of neutrophils and secretion of type-1 cytokines.

## MATERIALS AND METHODS

### Animals

Female C3H/HeN mice (10 weeks old) were purchased from Charles River Laboratories (Boston, MA, USA). Although the initial model of infection was developed using C3H-He mice (73) and C3H-HeJ was used often (69), we chose C3H-HeN expressing a fully functional tlr4 receptor for these studies. Mice were housed in the animal facility at the University of Tennessee Health Science Center (UTHSC). All procedures followed the NIH Guide for the Care and Use of Laboratory Animals and were approved by the UTHSC IACUC (#22-0400) and granted by the UTHSC Institutional Animal Care and Use Committee (IACUC).

### *Ixodes scapularis* maintenance and experimental challenge

*Borrelia burgdorferi*-infected *Ixodes scapularis* (black-legged) nymphal ticks were maintained under controlled temperature, humidity, and photoperiod as previously described (29). The founding cohort of infected nymphs was collected from multiple sites in Massachusetts in 2021. To maintain the colony, naïve C3H/HeN mice were infested with infected nymphs carrying 19 strains of Bb (OspC types A, B, C, D, E, F, Fa, Fb, G, H, I, J, K, L, M, N, O, W, X). Three weeks after the last day of challenge, seroconversion to Bb antigen was confirmed by pepVF ELISA (74) or Virablot (Viramed Biotech AG), and tick infectious rate was determined by Bb *flaB* qPCR using a subset of 10 ticks. The tick cohort infectious rate ranged from 5,000 to 35,000. Uninfected *I. scapularis* larvae (Oklahoma State University tick-rearing facility) were fed on Bb-infected mice. Fully engorged larvae were collected from cage bottoms into ventilated tubes, and a subset of 5–10 larvae per batch was tested for Bb by *flaB* qPCR to determine infection prevalence. Engorged larvae were held in an environmental chamber for ~6 weeks until molting to nymphs, then maintained for an additional ~6 weeks to allow diapause and onset of questing behavior (≈12 weeks post-engorgement).

Uninfected nymphs were produced using the same procedure except that larvae were fed on naïve mice. Questing infected or uninfected nymphs (*n* = 6–8 per mouse) were used to challenge experimental mice according to a laboratory protocol (27).

### Culture of *B. burgdorferi*

*B. burgdorferi* used for subcutaneous inoculation was cultured from an 11-strain (OspC types A, B, Ba, C, D, E, I, M, Q, W, X) New York field-derived isolate from 2008, maintained in our laboratory from a frozen stock (MS08). Spirochetes were grown in 4 mL of BSK-H complete medium (Sigma) at 34°C under microaerophilic conditions until mid-log phase. Cell density, morphology, and motility were monitored by dark-field microscopy using a Petroff–Hausser counting chamber, and cultures were adjusted with endotoxin-free PBS to a final concentration of 1 × 10⁵ spirochetes per 50 µL immediately prior to inoculation.

### DNA isolation and qPCR

DNA was extracted from mouse skin tissues using the NucleoSpin tissue genomic DNA extraction kit, following the manufacturer’s instructions. The isolated DNA was used for *B. burgdorferi* detection and quantification by qPCR. The quantification of *B. burgdorferi* was performed using the standard curve method, equivalent to the copy number of the *flaB* gene. Specific forward and reverse primers, along with TAMRA probes targeting fla (Eurofins, USA), were used. The standard curve was prepared by serially diluting Bb cultures ranging from 10^6^ to 10 spirochetes that were previously counted using dark field microscopy.

### Determination of infectiousness of Bb cultures and Ixodes ticks used

Infected nymphal ticks were used in concurrent studies, and proof of Bb dissemination to tissues is shown (27, 28). Multi-strain cultures of Bb were generated from an MS08 frozen stock, grown in BSK-H at 34°C, and 10^5^ Bb were inoculated into SC in the back of the head of C3H-HeN mice. Three weeks later, five nymphal ticks were allowed to feed on SC-infected mice and naturally fall off; 3 weeks after the last day of challenge, mice were euthanized and tissues collected for culture of Bb in BSK-H at 34°C. DNA was purified from the original MS08 culture, from ticks that fed on subcutaneously infected mice, and from heart culture from infected mice, and subjected to *ospC* type sequencing as described below.

### OspC amplicon generation, Illumina sequencing, and type quantification

DNA was extracted from three MS08-derived sample types: (i) the multi-strain 2008 *Borrelia burgdorferi* BSK-H culture (MS08), (ii) pooled *Ixodes scapularis n*ymphs infected by feeding on mice inoculated subcutaneously with MS08, and (iii) a *B. burgdorferi* culture established by placing a fragment of infected mouse heart into BSK medium, followed by outgrowth (heart-culture; MS08-derived). DNA was purified using the QIAamp DNA Blood & Tissue Kit (Qiagen, 69504). A 718-bp region of *ospC* was amplified using primers AATAAAAAGGAGGCACAAATTAATG (forward) and GTAACTGGAAAAATAAAGTCAATAT (reverse). Each 50-µL PCR contained 0.4 µM of each primer, 2.5 mM MgCl₂, 200 µM each dNTP (Thermo Scientific, R0191), 2.5 U Taq DNA polymerase with 1× Taq buffer (Thermo Scientific, EP0402), and 1 µL purified DNA template. Cycling conditions were 95°C for 4 min, followed by 36 cycles of 95°C for 30 s, 58°C for 30 s, and 72°C for 58 s, with a final extension at 72°C for 5 min. Libraries were prepared using the Nextera workflow with dual indexing and sequenced at the University of Tennessee Health Science Center Molecular Resource Center on an Illumina MiSeq (2 × 250 bp). Demultiplexed reads were aligned to an *ospC* reference panel representing types A, B, C, etc. using Bowtie2, and the number of reads mapping to each *ospC* type reference was tabulated. Abundances are reported as raw mapped read counts.

### Proteome profile array

Cytokines and chemokines in mouse serum were quantified using the Proteome Profiler Mouse Cytokine Array Kit (R&D Systems, USA) following the manufacturer’s protocol with minor modifications (75). The array measures 21 chemokines: BLC/CXCL13, C5/C5a, G-CSF, I-309/CCL1, Eotaxin/CCL11, ICAM-1, IP-10/CXCL10, I-TAC/CXCL11, KC/CXCL1, M-CSF, JE/MCP-1/CCL2, MCP-5/CCL12, MIG/CXCL9, MIP-1α/CCL3, MIP-1β/CCL4, MIP-2/CXCL2, RANTES/CCL5, SDF-1/CXCL12, TARC/CCL17, TIMP-1, and TREM-1. The 19 cytokine panel included TNFα, IFNγ, IL-1α, IL-1β, IL-1ra, IL-2, IL-3, IL-4, IL-5, IL-6, IL-7, IL-10, IL-12, IL-12p70, IL-13, IL-16, IL-17, IL-23, and IL-27. Serum from each group was pooled (~100 µL per mouse, *n* = 3) and applied to individual array membranes. Membranes were first blocked for 1 h with the supplied buffer, then incubated for 1 h at room temperature with a cocktail of 40 biotinylated detection antibodies. Samples were transferred to nitrocellulose membranes and incubated overnight at 4°C on a rocker. Each membrane contains duplicate capture-antibody spots for each analyte. After three washes, membranes were incubated with streptavidin–HRP and developed with chemiluminescent substrate. Multiple exposures were captured using a ChemiDoc imaging system (Bio-Rad), and mean pixel density was quantified with QuickSpot software (Ideal Eyes Systems, USA). Data were plotted in GraphPad Prism. Significant differences are determined at >0.58 log_2_ fold change, equivalent to 1.5 fold change.

### Flow cytometry

Single-cell suspensions from spleen and skin were prepared as previously described (75–77). Live/dead cell staining was performed (AO/PI, Luna, Logos Biosystems), and ~10^6^ cells per well were stained with fluorochrome-conjugated antibodies (Table S1). Cells were blocked with anti-mouse CD16/32 (1:50, 15–20 min, on ice), stained for 30 min at 4°C in the dark, washed, fixed with 4% paraformaldehyde, and resuspended in staining buffer. Data were acquired on a Bio-Rad ZE5 Cell Analyzer and analyzed using FlowJo (BD Biosciences). Gating strategies are shown in Fig. S2 and S3.

### How to interpret the immune cell proportion data

Populations of immune cells or its precursors in skin (the port of entry of *B. burgdorferi*) or in the secondary lymphoid organ (spleen) at any given time will be up, down, or not different than naïve control depending on their ultimate function and if they are resident or recruited cells (differentiated phagocytic/antigen-presenting cells, migratory precursors, migratory phagocytic/APC), with the nuance that differentiated cells may enter and exit the tissue to execute their phagocytic/killing/APC functions and undergo cell death after executing phagocytic/clearance functions. Flow cytometry results are reported as the percentage of total live cells within each gated population for skin and spleen. Because these values represent relative tissue composition rather than absolute cell numbers, changes relative to naïve/PBS controls can reflect recruitment into the tissue, egress from the tissue (e.g., migration to draining lymph nodes), and/or cell death after activation/effector function. Accordingly, an increase in one population may coincide with decreases in others without implying proportional changes in total cellularity.

### Generation of log₂ fold-change heatmaps

Serum chemokines and cytokines were quantified using the Proteome Profiler Mouse Cytokine Array as described above. For each analyte, mean pixel intensity (duplicate spots) was used to compute fold change relative to PBS controls (PbsSC) for each group (BbSC, Tick+Bb, Tick–Bb). Fold-change values were log₂-transformed such that 0 represents the PBS baseline and positive/negative values represent increased/decreased signal.

### Sample size and power

For two-group comparisons with equal group sizes, *n* = 6 mice per group provides ~80% power (α = 0.05, two-sided) to detect standardized differences of ~1.6 SD (Cohen’s d ≈ 1.6).

### Statistical analysis

The statistical analysis for this study was conducted using a one-way ANOVA test, and multiple comparisons between groups were analyzed with Tukey’s correction. Graphs were plotted using GraphPad Prism software, and a value of *P* <0.05 was considered statistically significant.

## Data Availability

The original contributions presented in the investigation are included in the article. Supplemental files and source files contain all the raw, processed, and supporting data. We will make data fully available and without restriction upon request.

## SUPPLEMENTAL MATERIAL

Figure S1 mbio.03971-25-s0001.tiff Disruption of *B. burgdorferi ospC* types across MS08-derived samples.

Figure S2 mbio.03971-25-s0002.tif Gating strategy for skin flow cytometry.

Figure S3 mbio.03971-25-s0003.tif Gating strategy for spleen flow cytometry.

Table S1 mbio.03971-25-s0004.docx Antibodies and fluorophores used for flow cytometry.

ASM does not own the copyrights to Supplemental Material that may be linked to, or accessed through, an article. The authors have granted ASM a non-exclusive, world-wide license to publish the Supplemental Material files. Please contact the corresponding author directly for reuse.
