# Supplementary figures and images for: Innate immune responses to Borrelia burgdorferi during tick-feeding: mechanistic insights relevant to Lyme disease
Source: mBio. 2026 Apr 20;17(5):e03971-25. doi: 10.1128/mbio.03971-25 (PMC13170348; doi:10.1128/mbio.03971-25)

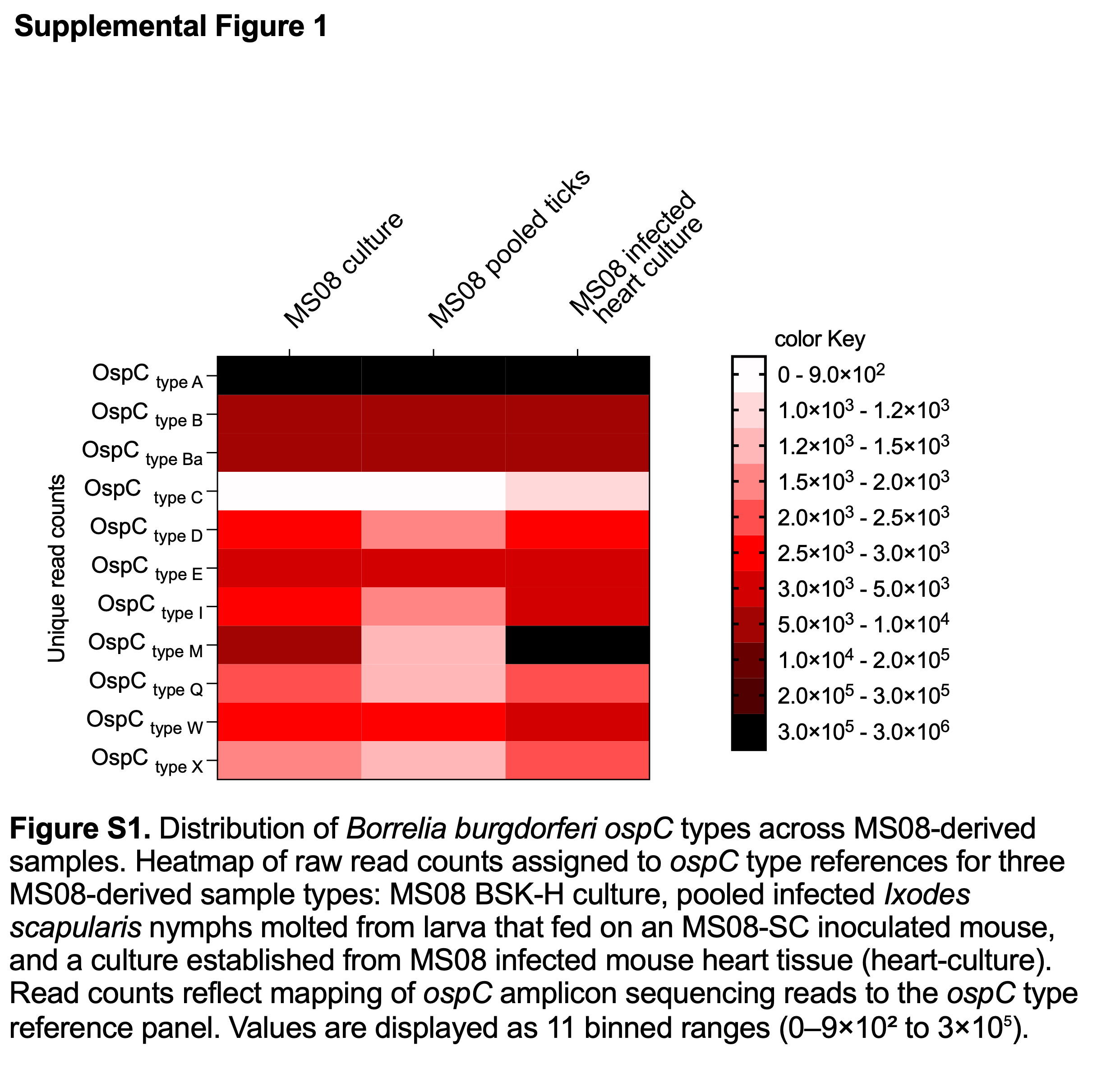

Supplement: Figure S1 — Disruption of B. burgdorferi ospC types across MS08-derived samples. [file mbio.03971-25-s0001.tiff]

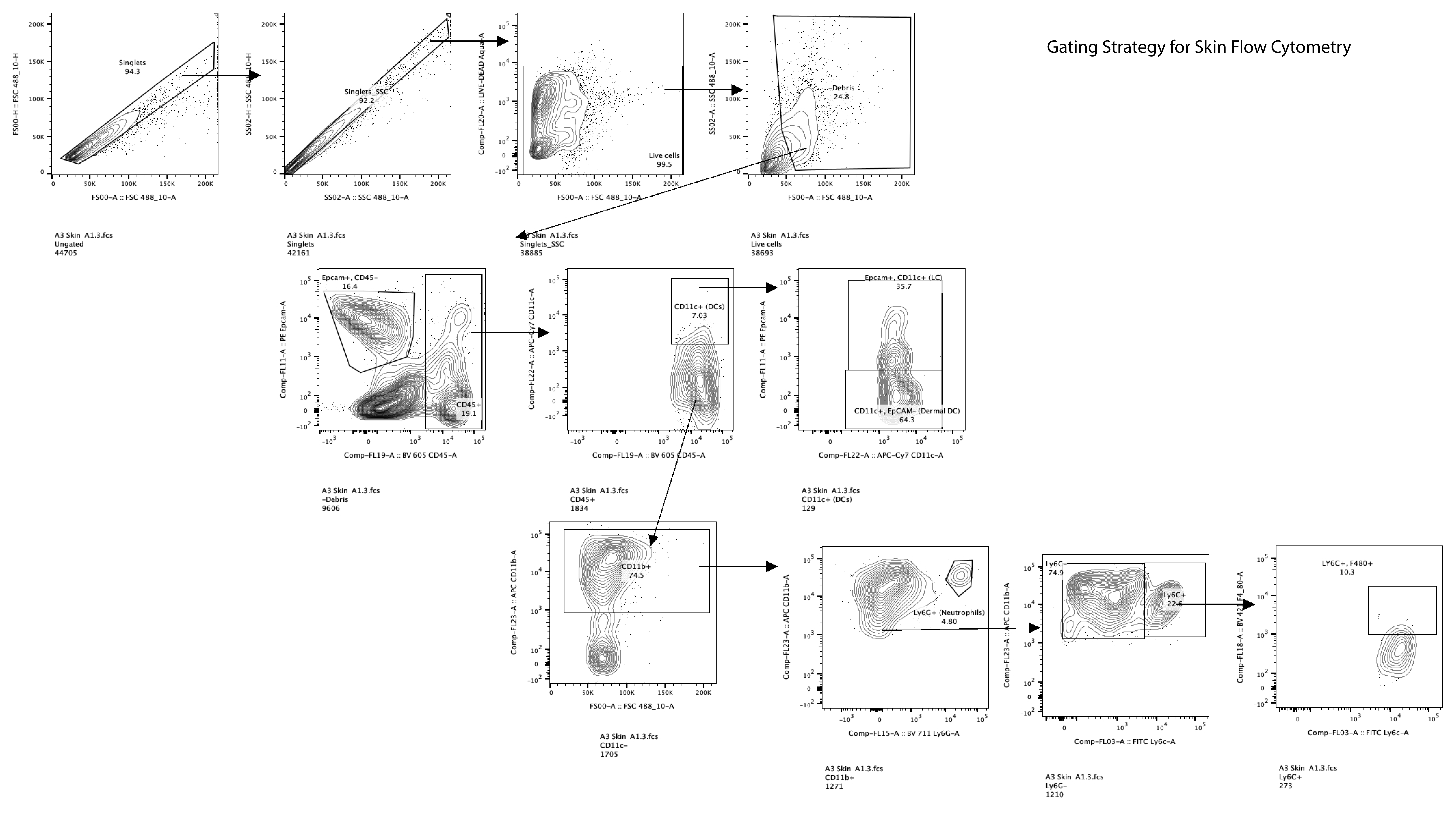

Supplement: Figure S2 — Gating strategy for skin flow cytometry. [file mbio.03971-25-s0002.tif]

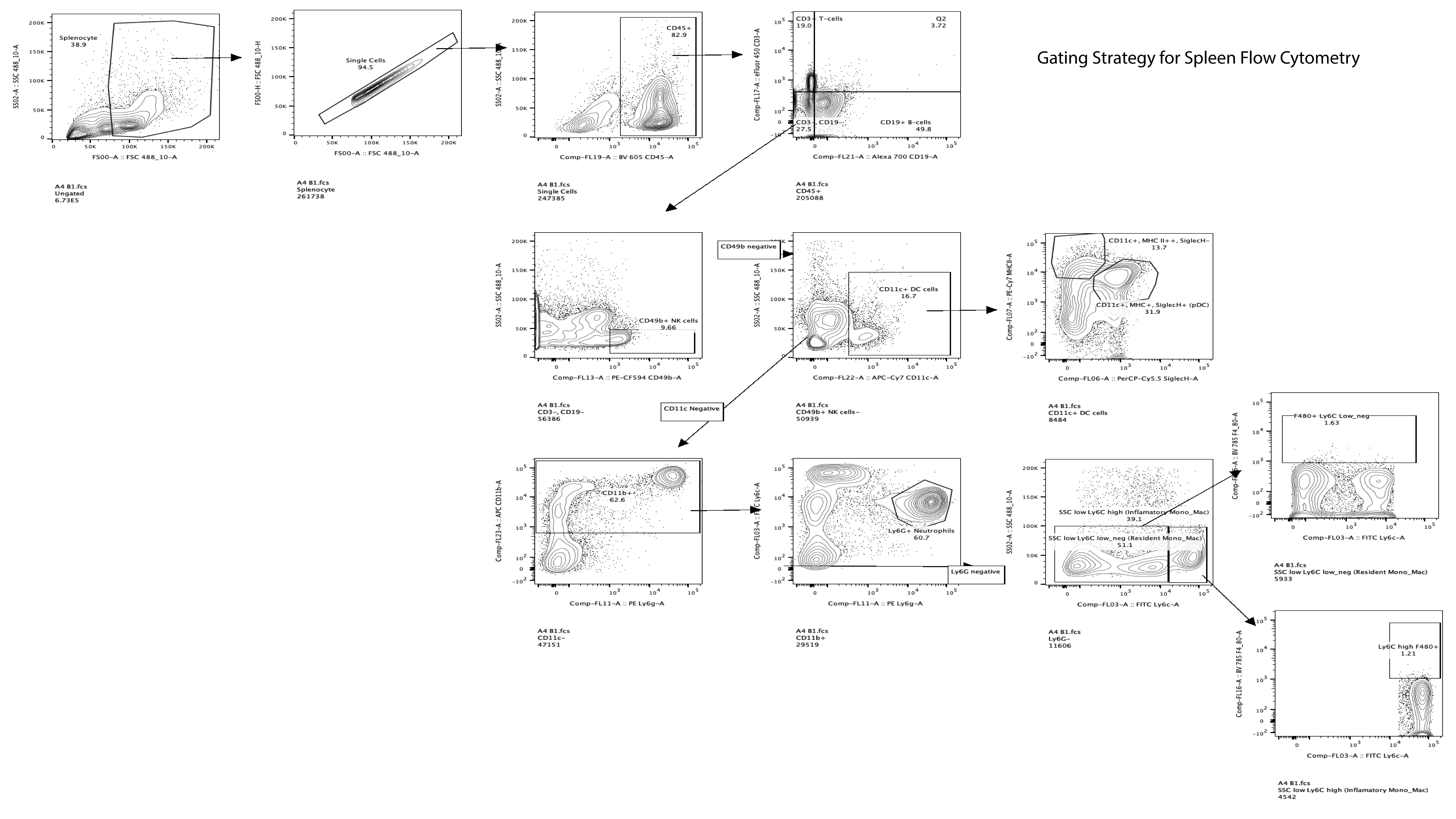

Supplement: Figure S3 — Gating strategy for spleen flow cytometry. [file mbio.03971-25-s0003.tif]
