# Supplementary material for: Innate immune responses to Borrelia burgdorferi during tick-feeding: mechanistic insights relevant to Lyme disease
Source: mBio. 2026 Apr 20;17(5):e03971-25. doi: 10.1128/mbio.03971-25 (PMC13170348; doi:10.1128/mbio.03971-25)
Supplement: Table S1 — Antibodies and fluorophores used for flow cytometry. [file mbio.03971-25-s0004.docx]

**Supplementary Information**

**List of antibodies used in Flow Cytometry.**

| **Marker** | **Fluorophore** | **Company** |
| --- | --- | --- |
| CD16/32 | Purified blocking antibody | Biolegend |
| CD45 | Brilliant Violet 605 | Biolegend |
| EpCAM | PE | Biolegend |
| Siglec H | PerCP-Cy5.5 | Biolegend |
| CD49b | PE Dazzle 594 | eBiosciences |
| F4/80 | Brilliant Violet 421 | Biolegend |
| CD11b | APC | TONBO Biosciences |
| CD11c | APC-Cy7 | TONBO Biosciences |
| Ly6C | FITC | Biolegend |
| Ly6G | Alexa 711 | Biolegend |
| MHC-II | PE-Cy7 | Biolegend |
| Live-dead | Zombie Aqua | Biolegend |
| CD3 | eFluor 450 | TONBO Biosciences |
| CD19 | Alexa Fluor 700 | Biolegend |
| F4/80 | BV785 | Biolegend |
| Ly6G | PE | Biolegend |

**Supplementary Table 1. Antibodies and fluorophores used for flow-cytometry** List of primary antibodies, fluorophore conjugates, and commercial sources employed for skin and spleen single-cell staining prior to flow cytometry. Blocking antibody (CD16/32) was applied before surface staining; live/dead discrimination was performed with Zombie Aqua.
